# Functional Characterization of Two scFv-Fc Antibodies from an HIV Controller Selected on Soluble HIV-1 Env Complexes: A Neutralizing V3- and a Trimer-Specific gp41 Antibody

**DOI:** 10.1371/journal.pone.0097478

**Published:** 2014-05-14

**Authors:** Maria Trott, Svenja Weiß, Sascha Antoni, Joachim Koch, Hagen von Briesen, Michael Hust, Ursula Dietrich

**Affiliations:** 1 Georg-Speyer-Haus, Institute for Tumor Biology and Experimental Therapy, Frankfurt, Germany; 2 HIV Specimen Cryorepository (HSC) at Fraunhofer Institute of Biomedical Engineering, St. Ingbert, Germany; 3 Technische Universität Braunschweig, Institute of Biochemistry, Biotechnology and Bioinformatics, Braunschweig, Germany; German Primate Center, Germany

## Abstract

HIV neutralizing antibodies (nAbs) represent an important tool in view of prophylactic and therapeutic applications for HIV-1 infection. Patients chronically infected by HIV-1 represent a valuable source for nAbs. HIV controllers, including long-term non-progressors (LTNP) and elite controllers (EC), represent an interesting subgroup in this regard, as here nAbs can develop over time in a rather healthy immune system and in the absence of any therapeutic selection pressure. In this study, we characterized two particular antibodies that were selected as scFv antibody fragments from a phage immune library generated from an LTNP with HIV neutralizing antibodies in his plasma. The phage library was screened on recombinant soluble gp140 envelope (Env) proteins. Sequencing the selected peptide inserts revealed two major classes of antibody sequences. Binding analysis of the corresponding scFv-Fc derivatives to various trimeric and monomeric Env constructs as well as to peptide arrays showed that one class, represented by monoclonal antibody (mAb) A2, specifically recognizes an epitope localized in the pocket binding domain of the C heptad repeat (CHR) in the ectodomain of gp41, but only in the trimeric context. Thus, this antibody represents an interesting tool for trimer identification. MAb A7, representing the second class, binds to structural elements of the third variable loop V3 and neutralizes tier 1 and tier 2 HIV-1 isolates of different subtypes with matching critical amino acids in the linear epitope sequence. In conclusion, HIV controllers are a valuable source for the selection of functionally interesting antibodies that can be selected on soluble gp140 proteins with properties from the native envelope spike.

## Introduction

NAbs against HIV-1 have been associated with protective immunity in numerous animal studies including the simian/human immunodeficiency virus (SHIV) macaque model [1]–[9] and transgenic humanized mice [10]–[14]. Very recently, broadly neutralizing antibodies were shown to even contribute to the control of viremia in macaques chronically infected by HIV-1 in a therapeutic setting [15], [16]. Furthermore, a number of broadly neutralizing monoclonal Abs (bnmAbs) targeting critical epitopes involved in HIV-1 entry have been isolated from patients with chronic HIV-1 infection (for review see [17]). The original set of four well characterized bnmAbs (b12 [18], 2F5 [19], 4E10 [20] and 2G12 [21], [22]) has been rapidly expanded during the last few years by direct cloning from Env-specific B-cells from chronically infected patients with bnAbs in plasma [23]–[29]. These much more potent mAbs essentially target four regions in the native trimeric Env spike, which is composed of three heterodimers of the outer envelope glycoprotein gp120, non-covalently linked to the transmembrane protein gp41: the CD4 binding site, variable loops V1/V2 and V3 in gp120 often implicating glycan structures, and the membrane proximal external region (MPER) in gp41 [17].

Characterization of the identified bnAbs often revealed unusual structural features as well as a high extent of mutations in the complementary determining regions (CDR) resulting from affinity maturation for evolving Env antigens [30]. Therefore, bnAbs need time to develop and, if present, are found in chronically infected patients after several years of infection [31]. HIV controllers are a promising source for the identification of nAbs, as here they have time to develop and mature over years in a rather uncompromised immune system and in the absence of therapeutic selection pressure. We previously identified LTNP and EC with neutralizing activity in plasma and dissected the humoral immune response based on phage libraries displaying short peptides [32] or longer HIV-1 Env fragments [33]. This allowed the identification of new linear and conformational epitopes able to induce neutralizing antibodies upon vaccination in mice [32], [34], [35].

In this study we aimed at characterizing Env-specific antibodies present in the plasma of one of our LTNPs. An immune scFv phage display library was generated from LTNP MH03 with nAbs in plasma and screened with soluble gp140, which contains gp120 and the ectodomain of gp41, i.e. lacking the transmembrane and the intracellular domains of gp41. Soluble gp140 molecules are able to form trimeric complexes that have previously been shown to better mimic native Env spikes than monomeric gp120 in terms of their antigenicity and immunogenicity [36]–[45]. Furthermore, gp140 immunogens structurally ressemble native trimeric envelope spikes by displaying a similar quaternary architecture [40]. Here we successfully used soluble gp140 from the ADA.C1 strain immobilized on beads to select and further characterize two interesting scFv antibody fragments form a phage library generated from LTNP MH03, a neutralizing V3-specific antibody and a trimer specific gp41 antibody.

## Materials and Methods

### Patient Sample and Ethics Statement

The patient sample MH03 is part of a study approved by the Ethics Commission of the section of Medicine of the Johann Wolfgang Goethe University of Frankfurt am Main, Germany. He gave his written consent. The patient has been infected with HIV-1 subtype B before 1986, had a viral load <50 copies/mL over years and CD4^+^ cell counts always >600/µL in the absence of any antiretroviral therapy (ART) [32]. The plasma of the patient had neutralizing activity against a panel of recombinant reporter viruses with *env* derived from primary HIV-1 strains on U87.CD4.CCR5 cells with reciprocal IC50 values ranging from 35 to 2,200 [32]. The patient did not have the *ccr5* Δ32 bp deletion, but carried the HLA-B*5701 allele overrepresented in LTNPs [46], [47].

### Generation of a scFv Phage Library from Patient MH03

Lymphocytes were isolated from patient MH03 by the Ficoll-Hypaque method (Pan Biotech, Aidenbach). mRNA was isolated using the QuickPrep mRNA Purification Kit (GE Healthcare, München). cDNA was synthesized using Superscript II (Invitrogen), random hexamer oligonucleotide primers and ∼100 ng mRNA. Antibody gene fragments were amplified as previously described and cloned into the pHAL14 vector [48]. After electroporation into electrocompetent XL1-Blue MRF’ cells, the colonies were resuspended in 40 ml 2×YT medium, aliquoted (1 mL) and either used for glycerol stocks by addition of 50% (v/v) glycerin in PBS or for packaging by superinfection with Hyperphage. 1.25×10^10^ scFv-vector containing bacterial cells were infected with 2.5×10^11^ Hyperphage particles [49], [50] and incubated at 37°C for 30 min without shaking followed by incubation at 37°C for 30 min with shaking (250 rpm). The scFv-phages in the bacterial supernatants were precipitated with 20% (w/v) PEG/2.5 M NaCl solution on ice and resuspended with PBS. Phage titration was done as described before [51]. The scFv display rate of the packaged libraries was controlled by 10% SDS-PAGE, Western-Blot and anti-pIII immunostain (mouse anti-pIII 1∶2000, goat anti-mouse IgG AP conjugate 1∶10000).

### scFv Selection on Soluble gp140 (Biopanning)

Lectin-purified ADA.C1 gp140 (5 µg for 5×10^7^ beads, see below) was immobilized on magnetic tosyl-activated beads by rotating o.n. at 4°C according to the instructions in the manual (Invitrogen). Coupling to beads was verified by FACS analysis with Env-specific antisera (FACS Calibur). For negative selection, beads were incubated with lectin-purified supernatant from CHO cells stably tranfected with the empty vector. For screenings, beads were washed five times with 0.25% (w/v) gelatin/0.5% Tween-20 (v/v) in PBS (PBSGT) and blocked at 37°C for 2 h with 0.25% gelatine in PBS (PBSG). 10^10^ scFv-phages were added to control beads for 2 h at room temperature. After magnetic separation, supernatants were removed, added to the ADA.C1 coated beads and incubated at 4°C o.n. under rotation. Beads were washed five times with PBSGT and the phages were eluted with trypsin (1% w/v) for 30 minutes at 37°C. The eluted phages (190 µL) were incubated with 20 mL of a freshly grown XL1-Blue MRF culture for 30 min at 37°C, centrifuged (5 min at 2,000 rpm), the pellet resuspended in 200 µL of medium and plated o.n. at 37°C on a large agar plate (2×YT/100 µg/mL glucose/50 mM ampicillin). The remaining 10 µL phage solution was used for titering. The bacterial clones were resuspended in 5 mL 2×YT medium, pelleted and either used for preparation of glycerol stocks or for packaging as described above. Infected cells were harvested by centrifugation for 10 min at 3,220×g and the pellet was resuspended in 30 mL 2×YT supplemented with 100 µg/mL ampicillin and 50 µg/mL kanamycin. Antibody phage were produced at 30°C and 250 rpm for 16 h. Cells were harvested by centrifugation for 10 min at 3,220×g. The supernatant containing the antibody phage (∼1×10^12^ cfu/mL) were directly used for the next panning round. Three selection rounds were performed.

### Colony PCR and Sequence Analysis of scFv-fragments

For genetic analysis of the selected scFv, the recombinant vectors were analyzed by colony PCR using the primers MHLacZ_f (5′-GGCTCGTATGTTGTGTGG-3′) and M96pIIIrev (5′- CCC TCA TAG TTA GCG TAA CG-3′). PCR-conditions were: 95°C for 30 sec, 51°C for 30 sec, 72°C for 2 min, with a total of 40 cycles. PCR products were purified (Macherey-Nagel Nucleo, Düren, Germany) and sequenced with the same primer pair on an ABI PRISM 310 Genetic Analyzer. Sequences were uploaded to the VBASE2 database (www.vbase2.org) to determine the composition of the gene fragments and the divergence from the respective germline sequences (germinality index) [52].

### scFv and scFv-Fc Expression and Purification

ScFv fragments were purified via His-tag affinity chromatography. The scFv encoding bacterial XL1-Blue MRF culture was induced with 50 µM IPTG for 3 h at 250 rpm at 30°C. The bacterial pellet was resuspended in ice-cold PE-Buffer (pH 8, 20% (w/v) sucrose, 50 mM Tris, 1 mM EDTA) and centrifuged at 20,000×g for 30 min. The supernatant was dialyzed against PBS (Servapor dialysing tube, 12–14). Chelating Sepharose Fast Flow (Amersham) was incubated with nickel sulfate solution (0.1 M NiSO_4_) and washed with PBS. Afterwards, the sepharose was incubated with the dialyzed scFv supernatant at 4°C for 30 min. After several washing steps, scFv were eluted with 0.1 M EDTA and dialyzed against PBS at 4°C o.n.

ScFv were cloned into the pCMV-hIgG1-Fc-XP vector [53] to express scFv-Fc antibodies and were analyzed by PCR with primers pCMVfor (5′-CGC AAA TGG GCG GTA GGC GTG-3) and pCMVrev (5′-CCA GGA GTT CAG GTG CTG-3′). ScFv-Fc were expressed in 293T cells after transient transfection with 10 µg/plate scFv-Fc encoding vectors. 293T cell supernatants were incubated with protein A agarose (Pierce), eluted in several fractions with elution buffer (0.1 M citric acid, 0.1 M Tri-sodium citrate, pH 2.5) and neutralized with 2 M Tris, pH 9. Pooled fractions were centrifuged and concentrated with 30 kDa cut-off amicons (Millipore).

### Expression and Purification of Soluble gp140 Env from HIV-1 ADA.C1

CHO-Lec3.2.8.1 cells stably expressing HIV-1 ADA.C1 gp140 were obtained from Ellis Reinherz [54]. Here, soluble gp140 is expressed from the pEE14 vector (Lonza) containing a Kozak sequence and the tissue plasminogen activator (tPA) leader peptide upstream of the gp140 sequence, which consists of the entire gp120 ADA (1–477) and the gp41 ectodomain (478–632). Additionaly, a mutation (R477S) inhibits furin cleavage of gp120 and truncated gp41. Gp140 expression and purification was performed as previously described [54]. Briefly, 5×10^6^ CHO cells stably transfected with the ADA.C1 encoding vector were grown in 40 mL GMEM medium (Sigma; 4 mL dialysed FCS without glutamine, 0.8 mL 50x nucleosides, 0.4 mL non essential amino acids, 0.4 mL L-glutamate/L-asparagine, 0.4 mL sodium pyruvate, 0.2 mL penicillin/streptomycin (0.5%), 50 µL MSX (L-methionin sulfoximin) until confluent and then cultured for 6 days in fresh medium with 4 mM sodium butyrate at 37°C and 5% CO_2_. Supernatants were harvested, centrifuged (20 min at 4,000 rpm), filtered (0.22 µm, Nunc) and a protease inhibitor cocktail (Sigma) was added. A column with *Galanthus nivalis* lectin agarose beads (Sigma) was used to bind glycosylated gp140 o.n. at 4°C at a flow rate of 0.1 mL/min. After washing, bound gp140 was eluted with 500 mM methyl-α-D-manno-pyranoside. Fractions were pooled and concentrated with 100 kDa cut-off centricons (Millipore). Protein concentration was measured by nanoDrop.

### Western Blot

Lectin-purified gp140 ADA.C1 was separated by 8% SDS-PAGE in loading buffer without DTT for 1 h at 130 V. After transfer onto a nitrocellulose membrane and blocking with 5% MPBST for 1 h at room temperature, the membrane was incubated with the primary antibody (1∶10,000 dilution in 5% MPBST) o.n. at 4°C. After 3 washes with PBST, the secondary antibody was added (1∶5,000 dilution in 5% MPBST) for 1 h at room temperature. Detection was performed with the ECL Western blot detection kit (Thermo Scientific).

### Immunoprecipitation

5 µg of purified scFv antibody fragments were amino-linked to 20 µL activated agarose beads and washed according to the manufacturer’s protocol (Thermo Scientific). Beads were incubated with 1000 µg lectin-purified ADA.C1 for 24 h at 4°C with rocking. After washing, the ADA.C1 antigen was eluted in several fractions with glycin/HCl and immediately neutralized with 1 M Tris, pH 9.5. Fractions from washing and elution steps were resolved on 8% SDS-PAGE and analyzed by Western blot. HIV-positive sera and the Env trimer-specific Md-1 antibody were used for detection.

### ELISA

96 well plates (Greiner) were coated with 200 ng lectin-purified gp140 protein/well in PBS o.n. at 4°C. After washing with 3 ml/well 0.5% Tween 20 in PBS (PBST), the wells were blocked with 5% skimmed milk powder PBST (MPBST) for 2 h at 37°C. After washing again as above, plates were incubated with antibody (1∶2,000 dilution in 5% MPBST) o.n. at 4°C. Again the plates were washed and incubated with HRP-conjugated secondary antibody (1∶1,000 dilution in 5% MPBST) for 1 h at room temperature. TMB substrate (KPL) was added at 100 µl/well and stopped with 1 N HCL (100 µl/well). Read out was at 450 nm and 650 nm as reference.

ELISA with solubilized and denatured gp41 was performed by pretreatment with 1% SDS and 50 mM DTT at 95°C for 5 min before coating on a 96 well plate. Washing, blocking and detection were performed as described above.

For phage ELISA, plates were coated and blocked as described above. 50 µL of phage supernatants from clones obtained after the third selection round and packaged in a 96 well plate formate were added o.n. at 4°C. Bound phages were detected with an HRP labelled anti-pVIII coat protein mAb (1∶5,000 dilution in 5% MPBST, GE Healthcare).

Reactivity of scFv with gp120 and gp140 was evaluated by incubating with 50 µl/well scFv supernatant from XL-1 scFv expressing cells. Washing, blocking and detection were performed as described above.

To determine potential autoreactivity of the scFv-Fc A2 and A7 antibodies we used commercial ELISAs from Biorad for Cardiolopin and for phosphatidylserine with the included controls according to the manufactureŕs instructions.

### Expression and Detection of Env on 293T Cells by FACS

293T cells were transfected with plasmids M118 encoding JR-FL Env [55]and pRC-CMV-Rev1b (Alex Balazs, Caltech) encoding Rev in HEPES/CaCl_2_ (Sigma). Two days after transfection, cells were washed and resuspended in PBS. 1.2×10^5^ cells were transfered to FACS vials, washed and stained with antibodies (final concentration 2.7 µg/mL) for 1 h in the dark at 4°C. After washing, the secondary antibody (goat anti-human IgG, PE labeled, Jackson) was added 1∶100 for 1 h at 4°C. Finally, cells were washed and resuspended in 200 µL 4% PFA for FACS analysis.

### Peptide Arrays

Overlapping peptides (18 mers, 9 mers and 7 mers) of the gp120 or the gp41 ectodomain were synthesized by Fmoc chemistry with activated PEG spacers on cellulose membranes. Automated parallel peptide synthesis was performed on a MultiPep RS instrument (Intavis) [56].

Membranes were rehydrated, washed and blocked with 5% (w/v) MPBST for 1 h at room temperature. This was followed by incubation with scFv-Fc antibody (1∶2,000 dilution in 5% MPBST) at 4°C o.n. and subsequent washing steps with 0.5% Tween-20/PBS and incubation with HRP-conjugated anti-human antibody (1∶1,000 dilution in 5% MPBST). Detection was performed with the SuperSignal West ECL Kit according to the manufactureŕs instructions (Thermo Scientific).

### Neutralization Assays

Neutralization studies were performed as described previously [33]. Briefly, standardized HIV-1 pseudoviruses were used on TZM-bl cells [57], [58]. Serial dilutions of scFv-Fc antibodies were preincubated for 90 minutes at 37°C with the pseudovirus stocks before addition to TZM-bl cells. Infection was performed in a 96-well plate for 48 h in triplicates. Relative luminescence units (RLU) were then determined in the cell lysates and the 50% inhibitory dose (IC50) was determined compared to virus only controls.

### Statistical Analysis

Graph Pad Prism 6 was used to perform graphs including mean and standard deviations (SD) (GraphPad Software Inc., La Jolla, CA). Two way ANOVA Tukeýs multiple comparison was used for statistical analysis.

## Results

### Generation of a scFv Phage Library from LTNP MH03

A scFv library was generated starting from peripheral blood mononuclear cells (PBMC) from patient MH03. RNA was reverse transcribed into cDNA, which served to amplify variable light (VL) regions from κ and λ chains and variable heavy (VH) regions by PCR. First the VL repertoire was cloned into the pHAL14 vector [48] followed by VH repertoire cloning. The library size resulted in 4×10^6^ independent clones for the κ library and 2×10^6^ independent clones for the λ library. After packaging with Hyperphage [49], [50] we obtained phage titers of 2.1×10^11^ for the κ library and 4.6×10^11^ for the λ library. Library packaging was controlled by anti-pIII immunoblot (data not shown).

### Expression and Characterization of Soluble ADA.C1 gp140 from Transfected CHO Cells as Target for Phage Display Screenings

**Figure 1:**
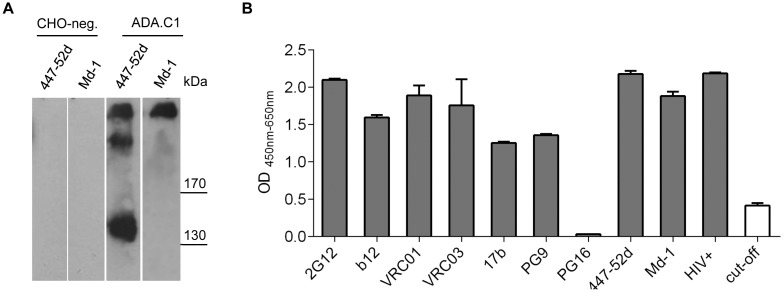
Characterization of soluble lectin purified ADA.C1 gp140 glycoproteins. (A) Western blot detecting ADA.C1 Env glycoprotein with anti V3 mAb 447-52D and trimer-specific mAb Md-1. CHO-neg corresponds to the supernatant of CHO cells transfected with empty vector and used as negative control for biopanning. (B) Binding of various mAbs directed against linear and conformational Env epitopes and HIV-positive sera to immobilized ADA.C1 determined by ELISA. Each sample was tested in duplicates and error bars represent standard deviations of the mean.

We expressed soluble ADA.C1 gp140 from CHO cells to be used as target for the selection of Env-specific scFv from LTNP MH03. We analyzed supernatants of transfected CHO cells by Western blot to determine the protomeric composition of the gp140 molecules present. Both, crude supernatants and lectin-purified gp140 contained monomeric, dimeric and trimeric gp140 (Fig. 1A). The trimeric version was specifically detected by mAb Md-1 [R.E. Myers, obtained from NIH AIDS reagent program]. The gp140 fractions were further analyzed for binding of a number of well characterized mAbs, including some with conformation-sensitive epitopes, by ELISA (Fig. 1B). The mixture of monomeric, dimeric and trimeric gp140 reacted with all linear and conformation-sensitive mAbs tested including PG9, but not with the related PG16, which very rarely binds to soluble gp140 by ELISA [59]. We therefore decided to use this antigenic gp140 mixture without further purification as target for our phage display screenings of the MH03 scFv library. Thus, epitopes occluded by trimerization would be accessible on the monomeric form and quaternary epitopes would be exposed on the trimeric form.

### Characterization of scFv from LTNP MH03 Selected with Immobilized gp140 ADA.C1

After one negative selection and three rounds of positive selection with soluble ADA.C1 gp140 immobilized on beads, scFv phages were tested by ELISA for specificity of gp140 binding. In parallel we tested the corresponding scFv fragments alone, i.e. outside the phage context. Six scFv showed strong specific reactivity with gp140, both in and outside the phage context (Fig. S1).

Sequencing of the six scFv vector inserts revealed two different sequences: scFv A7, B11, C8 and G5 were identical and scFv A2 and H7 were identical (Fig. S2). A further scFv, G4, only consisted of the variable region of the light chain and the C-terminal CDRH3 region of the heavy chain. This scFv was not analyzed functionally due to its poor expression rate. All scFv sequences were further analyzed by VBASE2 to determine the composition of immunoglobuline gene segments [60]. For the scFv A7 set the heavy chain was composed of IGHV5–51*01, IGHD4–17*01 and IGHJ3*01 or IGHJ3*02 and the light chain of IGLV3–1*01 and IGLJ2*01, IGLJ3*01 or IGLJ3*02. ScFv A2 consisted of the heavy chain IGHV1–46*01, IGHD3–10*01inv and IGHJ5*02 and the light chain IGLV2–8*01 and IGLJ1*01. The light chain of scFv G4 contained IGLV3–21*03 and IGLJ2*01 or IGLJ3*01. Thus, different gene segments were used for the selected scFv except for the J segment of the scFv A7 set and scFv G4, which were identical. Based on the sequence analysis, scFvA7 and A2 do not show extensive affinity maturation features, which are often seen in bnAbs like extended HCDR3 loops or a high degree of affinity maturation. The HCDR3 length is 12 aa for scFv A7 and 13 aa for scFv A2. The germinality index, i.e the deviation from the corresponding germline sequences, was 83% and 85% for the heavy and light chain respectively of scFv A7 and 92% and 97% for VH and VL of scFv A2. Thus, scFv A7 shows a certain degree of affinity maturation, as scFv from naïve libraries usually show more than 95% identity to the germline sequences [52]. We analyzed complete scFvs further with respect to their binding properties for different Env constructs as well as for their neutralizing activities: scFv A7 as representative of the A7, B11, C8 and G5 set and scFv A2 as representative of the A2, H7 family.

### Binding Specificity of scFvs A2 and A7 for Different HIV-1 Env Constructs

**Figure 2:**
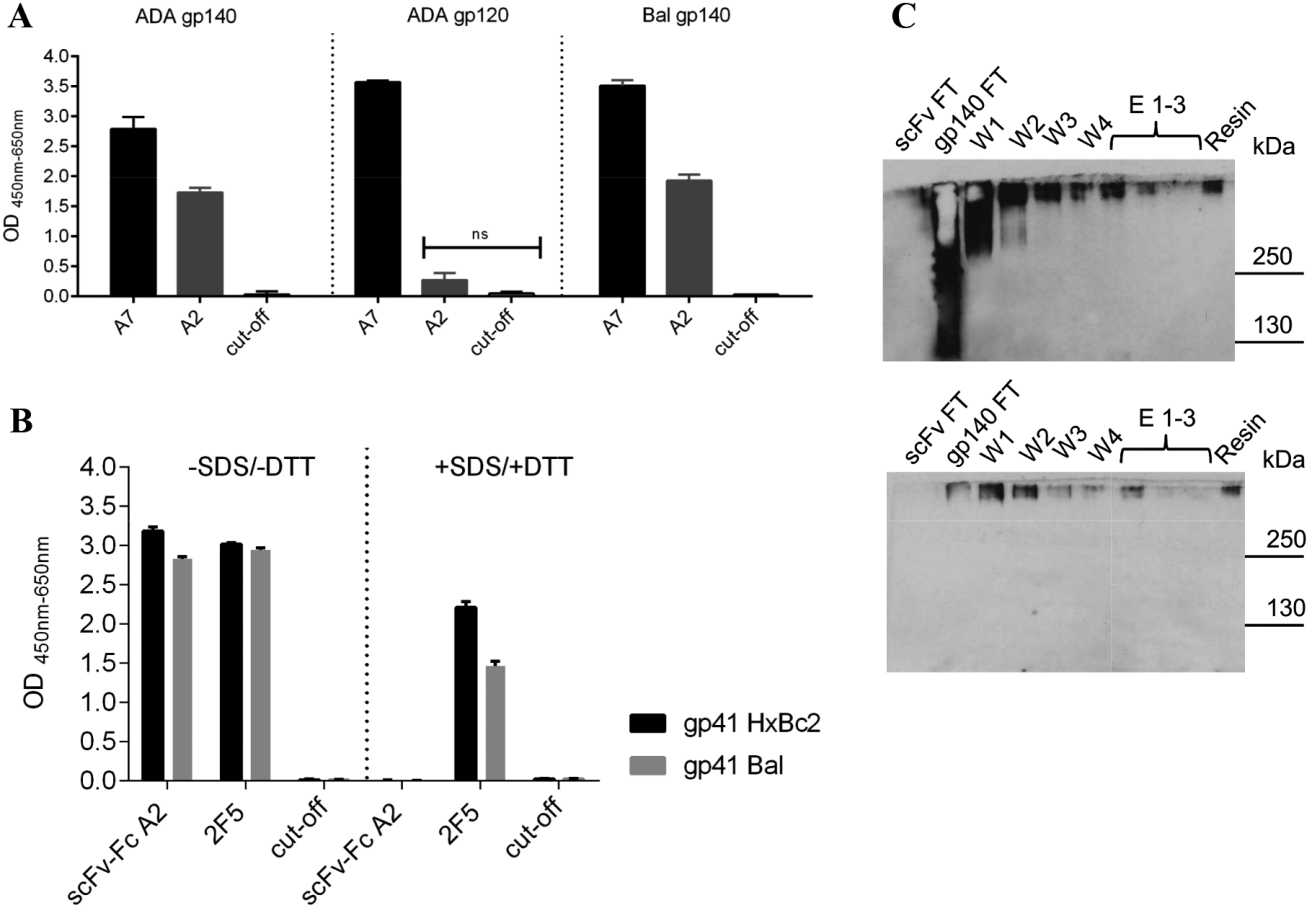
Recognition of Env proteins from different constructs by the selected scFv. (A) Binding of the two antibody classes scFv A7 and A2 to gp140 and gp120 Env proteins determined by ELISA. Detection was with anti c-myc antibody (300 ng/well) and HRP conjugated anti-mouse antibody (1∶1,000). (B) Reactivity of scFv-Fc A2 with SDS and DTT treated and non-treated gp41 Env proteins by ELISA. Each sample was tested in triplicates and error bars represent standard deviations of the mean. Significance analysis was performed with two way ANOVA Tukeýs multiple comparison test. (D) Immunoprecipitation of gp140 ADA.C1 protein from culture supernatants on scFv A2 coupled beads. Fractions of scFv A2 and gp140 flow through (FT), washing (W1-W4), elution (E1–E3) and control resin were analyzed by Western blot with HIV-positive serum (upper panel) and trimer-specific mAb Md-1 (lower panel).

We tested several gp120 and gp140 constructs for binding of scFvs A7 and A2 by ELISA (Fig. 2A). Interestingly, whereas scFv A7 reacted with monomeric gp120 as well as with the soluble gp140 molecules, scFv A2 only showed strong reactivity with gp140 Env. This could either mean that the epitope of scFv A2 is localized within the gp41 ectodomain, which is absent in gp120, or that this antibody recognizes trimeric Env present in the gp140 fractions. In order to distinguish between these possibilities, we performed ELISAs with gp41 proteins derived from two HIV-1 strains under native and denaturing conditions. Whereas mAb 2F5 recognizing a linear epitope in the membrane proximal external region (MPER) of gp41 reacted both, with monomeric and trimeric Env, scFv A2 only recognized two different gp41 molecules under non-reducing conditions (Fig. 2B). Thus, scFv A2 specifically recognizes the trimeric version of gp41.

**Figure 3:**
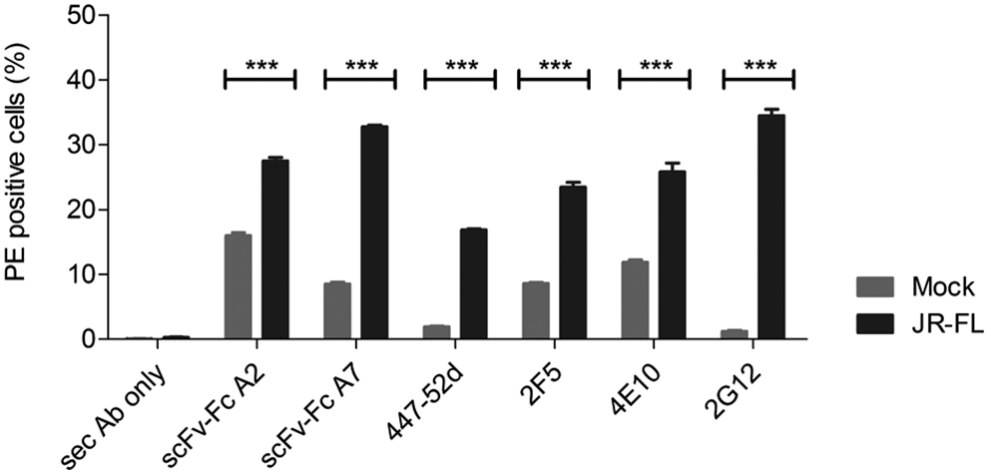
Recognition of native gp160 JR-FL Env on cells. Binding of scFv-Fc A2 and A7 as well as control antibodies to 293T cells transfected with JR-FL Env (black) or mock transfected (gray) by FACS analysis. Detection of bound antibodies was with a PE labeled secondary anti-human IgG antibody. Percent PE-positive cells are shown together with the secondary antibody only control. Each antibody was tested in triplicates and error bars represent the standard deviation of the mean.

To further prove the specificity of scFv A2 for trimeric gp140, we immunoprecipitated soluble gp140 ADA from culture supernatants with scFv A2 coupled to a resin. Fractions eluted from the resin and control fractions were analyzed by Western blots reacted with HIV-positive serum to identify all eluted gp140 forms and mAb Md-1 for trimer-specific recognition (Fig. 2C). Based on the reactivity with mAb Md-1, the eluted fractions E1–3 only contained the trimeric form of ADA gp140 proving the specificity of scFv A2 for immunoprecipitation of trimeric Env. Furthermore, the scFv-Fc A2 antibody also recognized native HIV-1 JR-FL Env on the surface of 293T cells transiently transfected with the corresponding gp160 plasmid (Fig. 3).

**Figure 4:**
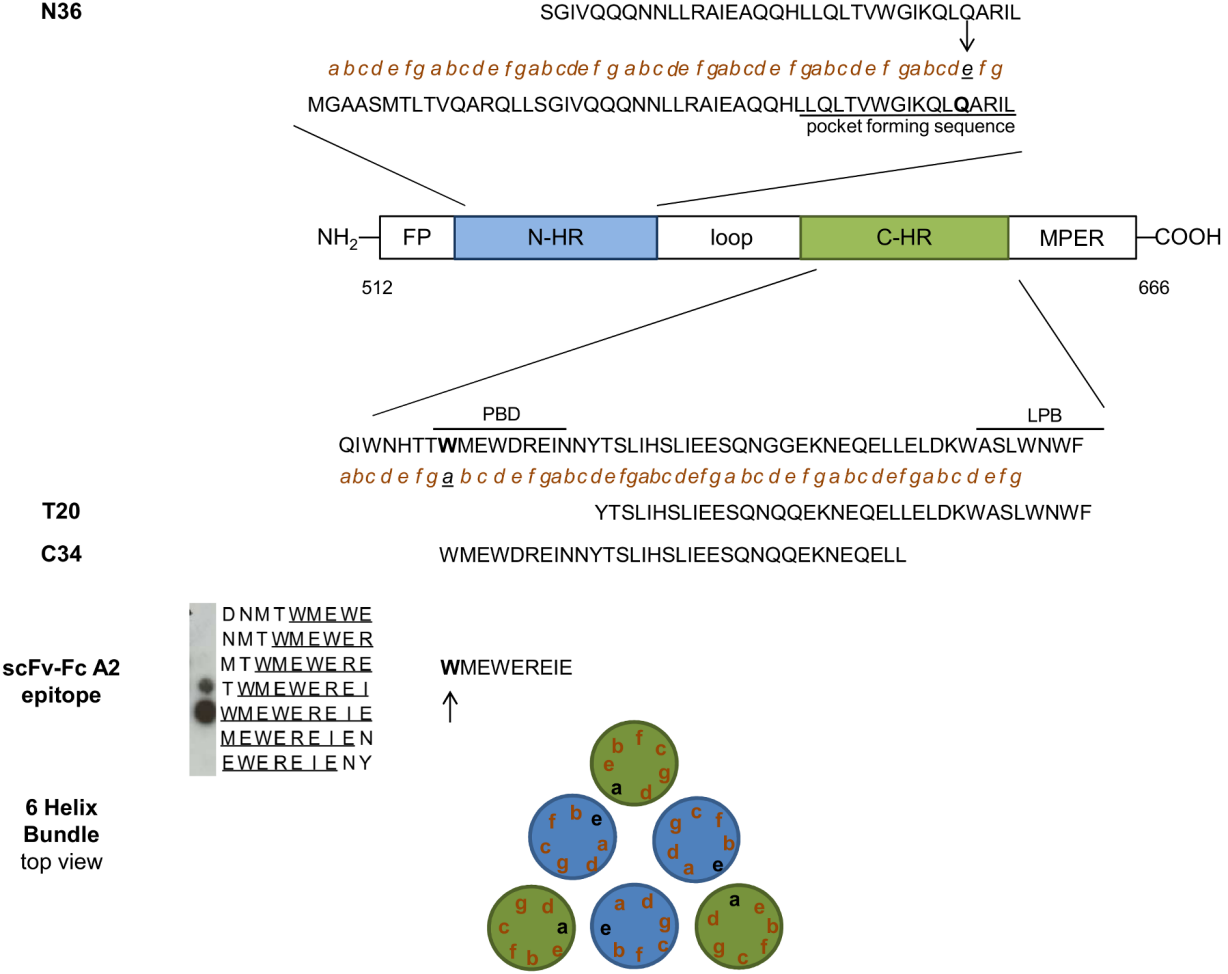
Epitope analysis of scFv-Fc A2. Schematic representation of functional domains of gp41: fusion peptide (FP), N-terminal heptad repeat (N-HR, in blue), C-terminal heptad repeat (C-HR, in green) and membrane proximal external region (MPER). The pocket-forming sequence in the N-HR domain and the pocket-binding domain (PBD) and lipid-binding domain (LBD) in the C-HR domain are underlined. Small letters “a”,”d” in C-HR and ”e” and “g” in N-HR mark interacting amino acid residues during 6 helix bundle formation. Sequences of N36, T20 and C34 petides as well as the scFv-Fc A2 epitope, as derived from the peptide array, are indicated. Further the schematic view of the gp41 6 helix bundle (top view) is depicted at the bottom.

The epitope recognized by scFv-Fc A2 was mapped based on the reactivity with a set of overlapping 18 mer peptides derived from the ectodomain of gp41 from the ADA strain on peptide arrays (Fig. 4). The epitope is located at the pocket-binding domain (PBD) of the C-terminal heptad repeat (CHR), which contacts a conserved hydrophobic pocket in the N-terminal heptad repeat (NHR) to stabilize the six-helix bundle formation enabling membrane fusion [61]. The epitope sequence “^1^WMEWEREIE^9^” corresponds to the N-terminus of the CHR derived C34 peptide that potently inhibits fusion (Fig. 4) [62], [63]. Interestingly, the epitope amino acid W1 corresponding to the “a” site in the alpa-helical wheel of CHR that interacts with the amino acid Q at the “e” site of the NHR in the six helix bundle [64] is critical for the recognition by scFvA2-Fc (Fig. 4). Amino acid I9, which also corresponds to an “a” site critical for interaction with the corresponding “e” site in NHR, is essential for recognition by scFv A2-Fc. Thus, six helix bundle formation may be a prerequisite for scFvA2-Fc binding enabling the critical juxtaposition of the key epitope in the trimeric context. Of note, the amino acid W1 is conserved in all HIV-1 gp41 sequences in the Los Alamos HIV sequence database 2012 (http://www.hiv.lanl.gov) and I9 is only very rarely substituted for “V” underlining the functional importance of these amino acids.

The breadth of reactivity of scFv-Fc A2 for a set of soluble gp140 proteins derived from different HIV-1 subtypes was analyzed by ELISA (Table 1). Seven of 15 gp140 Env proteins, all HIV-1 subtype B, reacted with scFvA2-Fc, 5 of which had only one amino acid exchange at the most C-terminal position of the epitope (E9->D or N). The two other reactive gp140 had an additional mutation, E3->Q or E5->D. All eight non reactive gp140 had 3 or more mutations in the scFvA2 epitope and correspond to non-B HIV-1 subtypes.

**Table 1:** ELISA reactivity of scFv-Fc A2 with a set of gp140 proteins.

clade	isolate	epitope	reactivity
consensus M	CON-S 140CF	WMEWEREIN	**+**
A	00MSA-4076 140CF	WMQWDKEVS	**−**
A	con-03 140CF	WLQWDKEIS	**−**
A	92RW020(VRC-A)140CFI	WLQWDKEIS	**−**
B	700010040_C9 140C	WMEWEREID	**+**
B	902114_B2_140C	WMQWEREID	**+**
B	624008_TA5_140C	WMEWEREID	**++**
B	con env-03 140CF	WMEWEREID	**+**
B	VRC_B_140CFI	WMEWDREIN	**+**
B	control ADA	WMEWEREIE	**++**
C	DU123.6 140CF	WMQWDREIS	**−**
C	1086C _140C	WMQWDREIN	**−**
C	089C 140C.pep	WMQWDREID	**−**
G	DRCBL 140CF	WIEWEREID	**−**
SIV	SIVcpzUS-1 140CF	WQEWDRKVR	**−**

−, +, ++ indicate no binding, binding and strong binding to scFv-Fc A2, respectively. Amino acid positions changed with respect to the original epitope WMEWEREIE on the peptide arrays are underlined.

**Figure 5:**
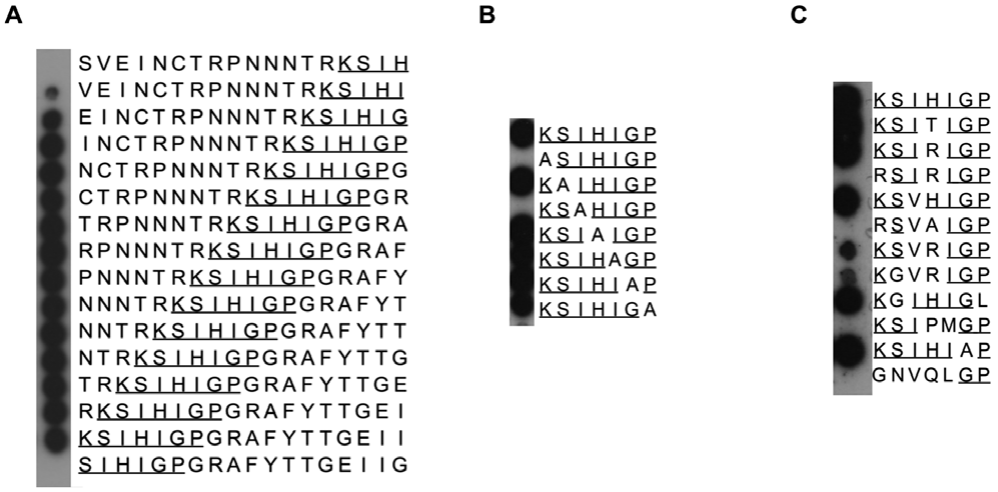
Epitope fine map of scFv-Fc A7 on 18 mer, and 7 mer peptide arrays. (A) 18 mer overlapping peptides of the ADA V3 epitope were incubated with scFv-Fc A7 identifying “KSIHIGP” (underlined) as core epitope. (B) Further fine mapping of the core epitope was analyzed via stepwise alanin substitutions in the 7 mer epitope. (C) Variations with 1, 2, 3 and 5 amino acids in the core epitope were analyzed for scFv-Fc A7 binding.

To identify the epitope of scFv-Fc A7, we analyzed its reactivity on peptide arrays with overlapping 18 mer peptides derived from gp120 of HIV-1ADA. This clearly localized the epitope in the V3 region (Fig. 5A). Fine mapping on arrays with V3 peptides with alanine modifications (Ala walk) or other amino acid substitutions identified the epitope for scFv A7 as “^1^K S I/V H/T/R I G/A P^7^” (Fig. 5B, C). Whereas the amino acid K1 is absolutely essential for the reactivity with scFv-Fc A7 and even cannot be substituted by the related amino acid arginine, more freedom is tolerated at the other positions of the epitope, in particular at position H4, which is often associated with antibody escape. This is also reflected in ELISAs with a set of soluble gp140 proteins from different HIV-1 subtypes, where the exchange of K1->R in the V3 epitope results in the absence of reactivity with scFv-Fc A7 (Table 2). Besides that, exchange of H4 for P in combination with I5 M (B.700010040_C9 140C) or a total of five mutations present in the SIVcpz epitope are not tolerated.

**Table 2:** ELISA reactivity of scFv-Fc A7 with a set of gp140 proteins.

clade	isolate	epitope	reactivity
consensus M	CON-S 140CF	KSIRIGP	**++**
A	00MSA-4076 140CF	KSVHIGP	**++**
A	con-03 140CF	KSIRIGP	**++**
A	92RW020(VRC-A)140CFI	KGVRIGP	**++**
B	700010040_C9 140C	KSIPMGP	**−**
B	902114_B2_140C	KSIHIAP	**++**
B	624008_TA5_140C	KGIHIGL	**++**
B	con env-03 140CF	KSIHIGP	**++**
B	VRC_B_140CFI	KSIHIGP	**++**
B	control ADA	KSIHIGP	**++**
C	DU123.6 140CF	KSIRIGP	**++**
C	1086C _140C	KSIRIGP	**++**
C	089C 140C.pep	RSIRIGP	**−**
G	DRCBL 140CF	RSVAIGP	**−**
SIV	SIVcpzUS-1 140CF	GNVQLGP	**−**

−, +, ++ indicate no binding, binding and strong binding to scFv-Fc A7, respectively. Amino acid positions changed with respect to the original epitope KSIHIGP on the peptide arrays are underlined.

**Figure 6:**
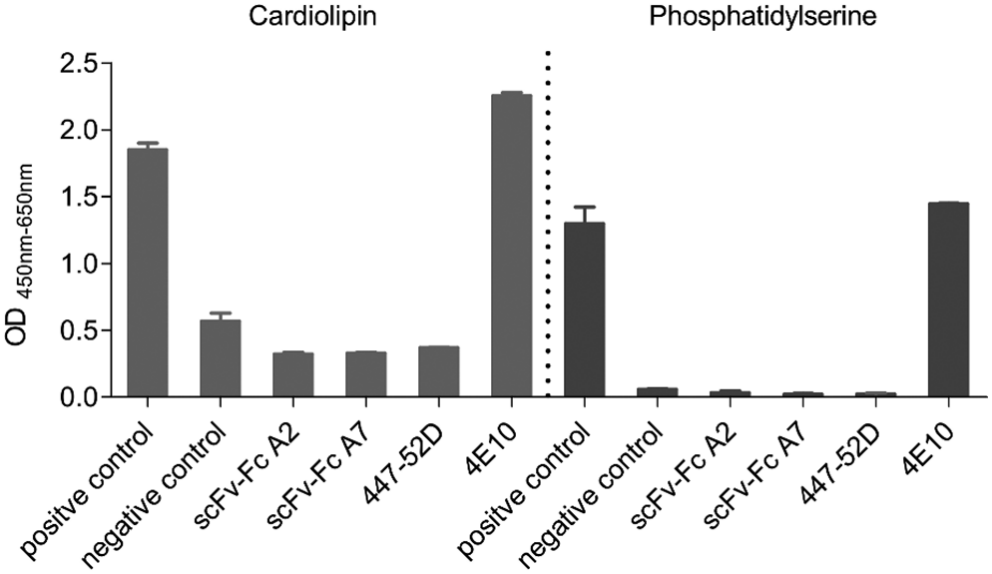
Analysis scFv-Fc A2 and A7 for autoreactivity against cardiolipin and phosphatidylserine. Autoreactivity of purified scFv-Fc A2 and A7 was analyzed by commercial ELISA (Bio-Rad) using the internal positive and negative control sera. mAb 4E10 was added as positive control and mAb 447-52D as negative control (each 100 µg/mL). Each sample was tested in duplicates and error bars represent the standard deviations of the mean.

Both, scFv-Fc A7 and A2 did not show autoreactivity with cardiolipin and phosphatidylserine by ELISA (Fig. 6).

### scFv-Fc A7 Neutralizes Strains with Conserved Epitope Sequences

The neutralizing capacity of scFv-Fc A7 was analyzed by *in vitro* neutralization assays using a panel of standardized Tier 1 and Tier 2 HIV-1 pseudovirions on Tzm-bl cells. Whereas scFv-Fc A2 had no neutralizing activity against this panel below 1 µM (data not shown), scFv-Fc A7 neutralized pseudovirions depending on the sequence of the A7 epitope (Table 3): two tier 1 strains with 100% epitope match (SS1196.1 and Bal26) were neutralized with an IC50 of 1.7 and 18 nM, respectively. The tier 2 strain TRJO4551 with 100% epitope match was neutralized with an IC50 of 626 nM. Mutations at position H4 did not alter much the neutralization sensitivity of tier 1 and tier 2 strains compared to strains with unmutated epitopes: tier 1 strain SF162.LS (H4->T) was neutralized with an IC50 of 1.1 nM and tier 2 strains RHPA4259.7 (H4->N) and CH110.2 (H4->N) were neutralized with IC50 of 784 nM and 645 nM, respectively. However, if in addition position K1 was mutated (K1->R in Tier 2 WITO4160) the IC50 increased to >1,000 nM. This underlines the importance of K1 for the A7 epitope, as already deduced from the peptide arrays.

**Table 3:** IC50 values of scFv-Fc A7 with a panel of tier 1 and tier 2 pseudoviruses in neutralization assay on tzm-bl cells.

Clade	Tier	Isolate	Epitope	IC50 (nM)	IC50 (µg/ml)
B	1	Bal.26	KSIHIGP	18	2.0
B	1	SS1196.1	KSIHIGP	1.7	0.19
B	1	SF162.LS	KSITIGP	1.1	0.12
A	1	MS208.A1	KSVRIGP	567	62.4
B	2	REJO454.67	KSIHIAP	333	36.6
B	2	WITO4160.33	RSINIGP	>1000	>110
B	2	TRJO4551.58	KSIHIGP	626	68.9
B	2	RHPA4259.7	KSINIGP	784	86.2
A	2	Q769.d22	KSIHLGP	359	39.5
A	2	Q23.17	KSIRIGP	783	86.1
C	2	DU156.12	KSVRIGP	530	58.3
C	2	DU422.1	KSVRIGP	725	79.7
BC	2	CH110.2	KSIRIGP	645	70.9

Pseudoviruses were preincubated with serial dilutions of scFv-Fc A7 before transduction of TZM-bl cells. Each sample was tested in triplicates and mean IC50 values are indicated.

## Discussion

Neutralizing antibodies are clearly associated with protection from HIV-1 infection [65], however, recent publications also suggest a potential beneficial role of these antibodies in chronic infection. A study by Klein and coworkers showed suppression of viral load by a combination of five very potent bnmAbs in a therapeutic setting in a humanized transgenic mouse model. Here, antibody treatment after established infection suppressed viremia below the detection limit for at least 60 days after the last treatment [66]. The therapeutic efficacy of neutralizing antibodies during chronic infection was very recently verified in two SHIV/macaque studies [15], [16]. These promising studies showed that bnmAbs can also act after infection by controlling the viremia and rose the question of a potential contribution of bnAbs in containing viremia in infected persons. In the past, several studies trying to answer this question led to controversial results. Although generally no correlation was found between the titers of nAbs and disease progression, a few studies suggest that neutralizing antibodies may contribute to the control of viremia: first, depletion of B-cells by rituximab in an HIV positive person with lymphoplasmacytoid lymphoma led to a reduction of autologous virus neutralizing antibodies accompanied by an increase in viral load [67]. After recovery of nAbs viral load dropped again indicating a clear inverse correlation of neutralizing antibody titers and viral load. Schmitz and coworkers also observed an inverse correlation between neutralizing antibody titers and viral load in rhesus monkeys infected by SIV after B-cell depletion following the acute phase of infection [68]. Concerning HIV-1 controllers, in a previous study we compared the neutralizing activities in plasma samples from a well defined group of LTNP and a control group of more recently infected persons with progressive disease, but with comparable viral load and CD4 count [32]. We detected statistically significant better neutralization titers against a set of viruses in the LTNP sera compared to the control group suggesting that bnAbs may potentially contribute to contain viremia in these particular LTNP, in which we could exclude virological and cellular factors as cause for non-progressive disease [46].

In this study, we now identified and characterized the Abs present in one of those LTNPs, MH03, by screening a phage library generated from this patient, which displayed his antibody repertoire in a scFv formate, with soluble gp140 derived from HIV-1ADA [54]. The lectin purified soluble gp140 fraction contained a mixture of monomeric, dimeric and trimeric Env allowing presentation of potential target epitopes in different contexts including the trimeric Env form mimicking best the native spike on HIV-1 virions. The presence of the trimeric form was proven by Western blot of HIV-1ADA.C1 gp140 reacted with the trimer-specific mAb Md-1 (Fig. 1A). The antigenic integrity of the gp140 constructs was reflected by good reactivity with a set of well characterized mAbs, some of those targeting conformational epitopes, by ELISA. With the exception of PG16, which very rarely reacts with soluble gp140 by ELISA [59], all other mAbs including the related PG9 showed very good reactivity with gp140ADA.C1. Thus, the soluble fractions of gp140ADA.C1 were used to select antibody fragments from LTNP MH03 from a scFv phage library generated from his B cells.

The selected scFv phages as well as the corresponding purified scFvs alone strongly bound to gp140ADA.C1 by ELISA. Based on the sequences of the most reactive scFv, these could be allocated to two groups, one represented by scFv A7 and the other by scFv A2. ScFv A2 recognizes an epitope localized in gp41, which is only recognized in the trimeric context. This was proven on Western blots of soluble gp140 from ADA.C1 immunoprecipitated with scFv-Fc A2, where only the trimeric form was eluted, as detected with human HIV-positive serum and trimer-specific Md-1 mAb (Fig. 2). ScFv-Fc A2 also recognized native Env expressed on the surface of cells transfected with HIV-1 JR-FL *env* constructs (Fig. 3). The high background observed with mock-transfected cells for scFv-Fc A2, but also for mAbs 2F5 and 4E10 known for their autoreactivity, prompted us to analyze the reactivity of our new antibodies with cardiolipin and phosphatidylserine by ELISA. No autoreactivity was detected in these ELISAs for scFv-Fc A2 and A7, whereas, as expected mAb 4E10 showed strong autoreactivity with phosphatidylserine and cardiolipin (Fig. 6). Thus, currently the high background reactivity observed here is still unknown.

The epitope sequence of scFv A2 comprising the amino acids ^1^ WMEWEREIE^9^ in the pocket binding domain of the CHR is not very well conserved except for the underlined hydrophobic aa residues, which dock into the deep pocket in NHR (Fig. 4) [64]. W1 is absolutely conserved among all published HIV-1 sequences and is critical for stabilizing the six-helix bundle formation necessary for fusion. The same is true for I8, which is only very rarely substituted by valine. W1 and I8 are absolutely essential for recognition by scFv-Fc A2 and represent aa in CHR making contacts to aa in NHR in the six-helix bundle structure. This may suggest that six-helix bundle formation is a prerequisite for scFv-Fc A2 binding and provide an explanation why this antibody is trimer-specific. However, other amino acids in the WMEWEREIE epitope are also important for antibody recognition, like M2 or E3, which are often substituted in non-B HIV-1 subtypes. Consequently, scFv-Fc A2 only recognized 7 of 15 gp140 constructs by ELISA, all of which were subtype B (Table 1). Thus, the trimeric context as well as linear motifs within the epitope seem to be important for recognition.

Interestingly, the scFv A2 epitope is contained within the epitope of mAb P2D2, which also recognizes gp140 in the trimeric context [69]. Of note, mAb P2D2 is a murine antibody cloned from hybridomas after immunization of mice with soluble gp140 from HIV-1 SF162. In contrast, scFv A2 was derived from a scFv library generated form one of our LTNPs. Although scFv-Fc A2 must not necessarily represent a naturally occurring human antibody due to potential unnatural pairing of heavy and light chains during the phage library construction, trimer-specific human mAbs have been identified previously, that target the C-HR domains in gp41 [70], [71]. Thus, such gp41 antibodies are also generated during natural infection induced by the native trimeric Env spike and are not antigenic “artefacts” selected on soluble gp140 immunogens. This is also underscored by the fact that scFv-Fc A2 also recognized native Env on transfected cells (Fig. 3). However, in contrast to the human mAb 126-6 and the murine mAb NC-1, which target cluster II regions in C-HR of gp41, scFv A2 targets a more N-terminal region in the PBD region. Due to the fact that the fusion process is common to all HIV-1 strains and that the pocket domain plays an important role in stabilizing the 6 helix bundle necessary for fusion, further investigation has to figure out whether antibodies against the PBD of gp41 may inhibit HIV-1 entry. In our study, scFv A2 did not show neutralizing activity against the tested isolates at concentrations below 1 µM and it also lacks major deviations from the germline sequence, which are often found in bnAbs. Of note, the murine mAb P2D2 with an epitope spanning the A2 epitope is also not neutralizing.

The epitope of scFv A7 could be mapped to the V3 region in gp120 based on its reactivity with HIV-1 Env peptide arrays. The immunodominant V3 region comprises conserved structural features despite variable amino acid composition, which is due to structural constraints imposed by its implication in coreceptor binding and virus entry [72]–[75]. Consequently, antibodies against V3 often show broad neutralizing activity across clades, when they target conformation-dependent epitopes [76]–[80]. Indeed scFv-Fc A7 showed potent neutralizing activity in the nanomolar range (IC50 <2 µg/mL) against Tier 1 strains. Against Tier 2 strains the neutralizing potency was much lower (IC50 between 36 and 110 µg/mL) depending on the mutations within the epitope (Table 3). Interestingly, the antibody gene fragments used in scFv A7 exactly correspond to those most abundantly found in neutralizing V3 antibodies, i.e. the variable heavy chain VH5-51 paired with the variable light chain VL3-1 [76]. The VH5-51 chain used in 18 of 51 (35%) V3 antibodies, in combination with a lambda chain (preferentially VL3-1 and VL1-47), defines a conserved antigenic structure in V3 which is recognized by these mAbs [81]. The antibody contact residues within this conserved antigenic structure in the V3 crown have been defined [81] and four of those are contained in the epitope of scFv A7 (K1, I3, H4 and I5). Except K1, which is localized in the N-terminal band of V3, the other contact residues in the scFv A7 epitope are localized in the N-terminal circlet next to the GPGR β-turn motif in the crown. Similarly, the corresponding contact residues in the CDR regions of VH5-51 and VL3-1 V3 antibodies identified in the Gorny study from 2011, which are conserved in the corresponding germline genes, are also found at the corresponding positions in our scFv A7 (marked in Fig. S2). Thus, scFv A7 belongs to a group of V3 mAbs recognizing structural elements N-terminal to the V3 crown and according to the epitope sequence recognized is most closely related to mAb 2257 [82], mAb 311-11D [83]and mAb 41148D [84], which are also neutralizing. These antibodies share most of their antibody gene segment composition (www.hiv.lanl.gov/content/immunology) and critical amino acids, which have been identified as important V3 contact residues ([81]; Fig. S2). Interestingly, this type of structural V3 antibodies is induced independently of the infecting HIV-1 subtype, as our scFv A7 is derived from an individual infected with HIV-1 subytpe B, whereas mAb 2257 originates from a patient from Cameroon infected with CRF02_AG.

In this study we could select two interesting scFv, A7 and A2, from LTNP MH03 on gp140 preparations from ADA.C1 containing a mixture of trimeric, dimeric and monomeric uncleaved Env proteins. It is remarkable, that scFv-Fc A7 recognizes an epitope in V3, which is highly similar to a mimotope that we previously selected from a random peptide phage library with IgG from the same patient (6x KXXHXGP and 1x KXIXXGP). However, although scFv-Fc A7 shows neutralizing activity, it probably does not represent the entire neutralizing activity of the parental plasma. The fact that we “missed” additional nAbs may be due to the presence of different antigenic structures in our uncleaved gp140 preparations. Very recently soluble uncleaved gp140 trimers of the subtype A BG505 SOSIP.664 have been shown to be very heterogeneous and that cleavage is necessary to obtain homogeneous trimers that better mimic native spikes [85]. The cryo-EM and the crystal structure of these trimers have been recently elucidated as well, allowing new insights into trimer assembly and trimer-dependent epitopes [86], [87]. Thus, in the future cleaved well defined gp140 SOSIP constructs may be most appropriate for the selection of broadly neutralizing antibodies from patients or immunized animals.

The antibodies identified here are similar, but different, to related antibodies described previously recognizing either V3 or trimeric gp140 (see above) and may thus contribute to further elucidate structural features of their target epitopes and recognition. In particular scFv-Fc A2 targeting a conserved pocket binding domain in gp41, but only in HIV-1 subtype B, in the trimeric context may be useful to delineate subtype specific differences in this functionally important gp41 domain.

## Supporting Information

Figure S1 **Reactivity of the selected scFv-phages and the corresponding soluble scFv with ADA.C1 Env protein.** Binding of the selected phages A2 and A7 on ADA.C1 coated plates (200 ng/well) by ELISA. Cut-off represents control scFv-phage and scFv (D1.3 against lysozyme). ScFv-phages were detected with an HRP-conjugated anti-M13 antibody, whereas scFv were detected with a mouse anti c-myc antibody (300 ng/well) and a secondary anti-mouse HRP-conjugated antibody (1∶1,000).
(TIFF)

Figure S2 **Sequence alignment of the scFv selected from the LTNP MH03 phage library.** Alignments of the scFv sequences revealed three different antibody classes, one comprising A7, B11, C8 and G5, the second comprising A2 and H7, while the third (G4) lacks most of the variable heavy chain depicted by dashed lines and was not further evaluated due to low expression. Complementary determining regions (CDRs) of the heavy (H1, H2, H3) and light (L1, L2 and L3) chains are marked in grey boxes. The linker sequence is shown in blue. Red boxes mark common contact residues of VH5–51 anti-V3 antibodies in complex with V3 antibodies as described in [81].
(JPG)
