# Supplementary figures and images for: Functional Characterization of Two scFv-Fc Antibodies from an HIV Controller Selected on Soluble HIV-1 Env Complexes: A Neutralizing V3- and a Trimer-Specific gp41 Antibody
Source: PLoS One. 2014 May 14;9(5):e97478. doi: 10.1371/journal.pone.0097478 (PMC4020869; doi:10.1371/journal.pone.0097478)

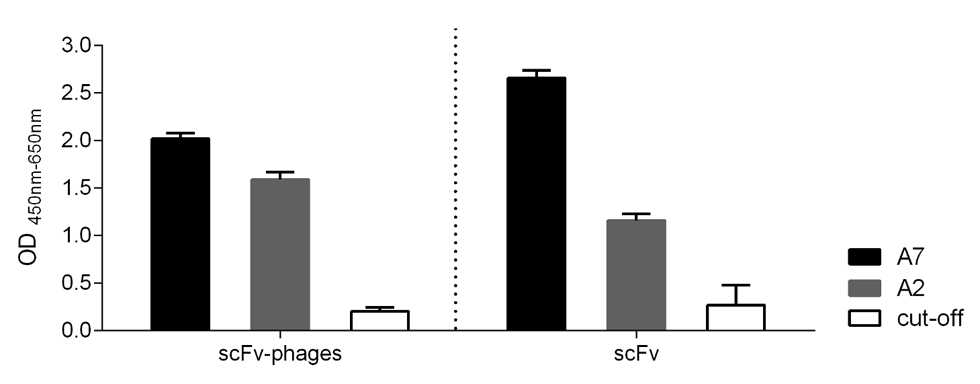

Supplement: Figure S1 — Reactivity of the selected scFv-phages and the corresponding soluble scFv with ADA.C1 Env protein. Binding of the selected phages A2 and A7 on ADA.C1 coated plates (200 ng/well) by ELISA. Cut-off represents control scFv-phage and scFv (D1.3 against lysozyme). ScFv-phages were detected with an HRP-conjugated anti-M13 antibody, whereas scFv were detected with a mouse anti c-myc antibody (300 ng/well) and a secondary anti-mouse HRP-conjugated antibody (1∶1,000). (TIFF) [file pone.0097478.s001.tiff]

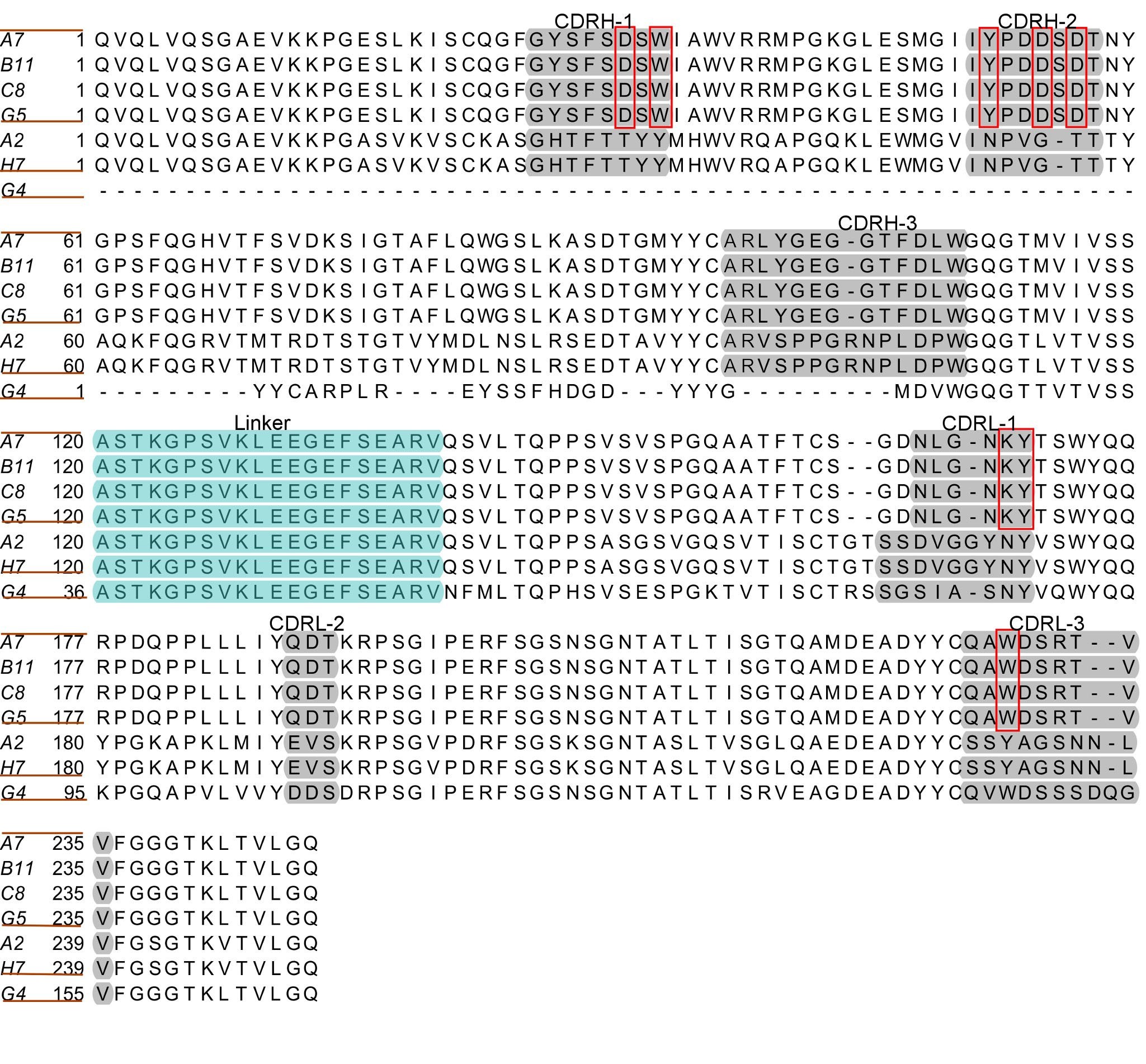

Supplement: Figure S2 — Sequence alignment of the scFv selected from the LTNP MH03 phage library. Alignments of the scFv sequences revealed three different antibody classes, one comprising A7, B11, C8 and G5, the second comprising A2 and H7, while the third (G4) lacks most of the variable heavy chain depicted by dashed lines and was not further evaluated due to low expression. Complementary determining regions (CDRs) of the heavy (H1, H2, H3) and light (L1, L2 and L3) chains are marked in grey boxes. The linker sequence is shown in blue. Red boxes mark common contact residues of VH5–51 anti-V3 antibodies in complex with V3 antibodies as described in [81]. (JPG) [file pone.0097478.s002.jpg]
